# Transcriptome profiling of soybean (*Glycine max*) roots challenged with pathogenic and non-pathogenic isolates of *Fusarium oxysporum*

**DOI:** 10.1186/s12864-015-2318-2

**Published:** 2015-12-21

**Authors:** Alessandra Lanubile, Usha K. Muppirala, Andrew J. Severin, Adriano Marocco, Gary P. Munkvold

**Affiliations:** Department of Sustainable Crop Production, Università Cattolica del Sacro Cuore, Via Emilia Parmense 84, 29122 Piacenza, Italy; Department of Plant Pathology and Microbiology, Iowa State University, 50011 Ames, IA USA; Genome Informatics Facility, Office of Biotechnology, Iowa State University, 50011 Ames, IA USA

**Keywords:** RNA-Seq analysis, *Fusarium oxysporum*, Plant defense, *Glycine max*

## Abstract

**Background:**

*Fusarium oxysporum* is one of the most common fungal pathogens causing soybean root rot and seedling blight in U.S.A. In a recent study, significant variation in aggressiveness was observed among isolates of *F. oxysporum* collected from roots in Iowa, ranging from highly pathogenic to weakly or non-pathogenic isolates.

**Results:**

We used RNA-seq analysis to investigate the molecular aspects of the interactions of a partially resistant soybean genotype with non-pathogenic/pathogenic isolates of *F. oxysporum* at 72 and 96 h post inoculation (hpi). Markedly different gene expression profiles were observed in response to the two isolates. A peak of highly differentially expressed genes (HDEGs) was triggered at 72 hpi in soybean roots and the number of HDEGs was about eight times higher in response to the pathogenic isolate compared to the non-pathogenic one (1,659 vs. 203 HDEGs, respectively). Furthermore, the magnitude of induction was much greater in response to the pathogenic isolate. This response included a stronger activation of defense-related genes, transcription factors, and genes involved in ethylene biosynthesis, secondary and sugar metabolism.

**Conclusions:**

The obtained data provide an important insight into the transcriptional responses of soybean-*F. oxysporum* interactions and illustrate the more drastic changes in the host transcriptome in response to the pathogenic isolate. These results may be useful in the developing new methods of broadening resistance of soybean to *F. oxysporum*, including the over-expression of key soybean genes.

**Electronic supplementary material:**

The online version of this article (doi:10.1186/s12864-015-2318-2) contains supplementary material, which is available to authorized users.

## Background

Soybean (*Glycine max* [L.] Merrill) is a major world crop and is the second most cultivated crop in the U.S.A. following maize. Soybean production contributes billions of dollars annually to the national economy, providing approximately 21 billion dollars in 2012 (United Nations Food and Agriculture Organization website). Limitations on maximum production are largely due to disease pressures that reduce yield. Several species of *Fusarium* have been associated with soybean, causing seed and seedling diseases, root rot, and vascular wilt [1–3]. *F. oxysporum* Schltdl. is a fungal soil-borne facultative parasite present worldwide [4] and is the most common species isolated from soybean roots in Iowa and other soybean-producing regions in North America [3, 5, 6]. *F. oxysporum* is known to consist of many “cryptic species” and, as such, it is often referred as the “*F. oxysporum* species complex” (FOSC). Recently, significant variation in aggressiveness was observed among isolates within the FOSC collected from soybean roots in Iowa [7, 8]. Some of these isolates caused severe root rot and dumping-off, other isolates were weakly pathogenic or non-pathogenic.

Management of soil-borne diseases like *Fusarium* root rot and wilt disease depends primarily on seed treatments and genetic resistance. Seed treatments are only effective during emergence and the seedling stages. Cultural practices can help in managing root rots, but these are often not adequate. Moreover, it seems clear that managing *Fusarium* root rot in the long-term will depend on improvements in molecular breeding for resistant genotypes [9].

Soybean resistance to other *Fusarium* species has been identified. Particularly high levels of resistance to *F. graminearum* have been found in the soybean cultivar Conrad, and putative QTL associated with resistance to *F. graminearum* have been detected [10, 11]. Previous work has identified QTL associated with resistance to sudden death syndrome (SDS), caused by *F. virguliforme* and other species, in Ripley [12] and Forrest [13] soybean genotypes. Progress in breeding for resistance will be improved through the analysis of new and consistent QTL for *Fusarium* root rot and wilt disease and by a deeper knowledge of genetic mechanisms underlying soybean-*F. oxysporum* interactions.

The availability of reference genome sequences and gene annotations for *G. max* and *F. oxysporum* has enabled us to study the molecular interactions between the host plant and its pathogen. Emerging massively parallel sequencing techniques allow the rapid acquisition of huge amounts of genomic or transcriptomic sequence data at relatively low costs [14]. To date, microarray techniques have been predominantly used for gene expression analysis particularly for well-studied model organisms for which typically high-quality gene annotation data were available. Compared with microarrays, RNA-Seq is known to have a wider dynamic range, higher technical reproducibility, and provide a better estimate of absolute expression levels [15, 16]. Genome-wide expression profiling of plants infected with *F. oxysporum* has been reported in several crop plant species, including melon [17], *Arabidopsis* [18], and banana [19]. However, little is known about transcriptional changes in soybean roots that have been infected by *F. oxysporum* and almost no attention has been paid to the study of differences in plant responses based on the pathogenicity of the infecting isolates. Most likely this is because of the scarce availability of isolates able to infect the same host while displaying a range of pathogenicity. This situation draws considerable interest in comparing root transcriptional responses between non-pathogenic and pathogenic *F. oxysporum* isolates.

To elucidate the comprehensive gene expression profiles for both *G. max* and *F. oxysporum*, we analysed the transcriptomes of plants and non-pathogenic and pathogenic fungi at 72 and 96 h post inoculation (hpi). Our aim was to characterize soybean genes that are differentially regulated by the host during infection by each pathogen, in order to identify new potential resistance mechanisms and candidate genes that have not previously been shown to play a role in defense. At the same time, we tried to strengthen our knowledge concerning novel details of infection by using two different pathogenic and non-pathogenic isolates of *F. oxysporum*. The results showed markedly different gene expression profiles in different host-pathogen combinations. A peak of HDEGs was observed at 72 hpi in soybean roots in response to both isolates and the number of HDEGs was about eight times higher for the pathogenic isolate compared to the non-pathogenic one. Furthermore, not only the number of genes, but also the magnitude of induction was much greater in response to the pathogenic isolate. These findings generated useful resources for the soybean research community and provided more insights into the understanding of soybean-*F. oxysporum* interactions.

## Results

### Quantification of fungal growth and disease severity evaluation in soybean roots

To determine the appropriate time points for the investigation of soybean transcriptome profiles following the FO36 (non-pathogenic) and FO40 (pathogenic) *F. oxysporum* isolates inoculation, quantification of the fungal *translation elongation factor* 1 *alpha* (*tef*1*α*) gene by quantitative PCR (qPCR) was carried out in this study. Infection progression was monitored in inoculated roots of partially resistant soybean genotype Forrest over a time course of seven days. The *tef*1*α* gene was detected in samples collected 48 h post inoculation (hpi) through 168 hpi with both isolates. Two-factor analysis of variance (ANOVA) revealed significant (P ≤ 0.001) differences between the times of sampling (48, 72, 96 and 168 hpi) in the observed means for the fungal *tef*1*α* DNA quantity and between the two treatments (non-pathogenic and pathogenic inoculated samples), and their interactions (Additional file 1: Table S1).

**Fig. 1:**
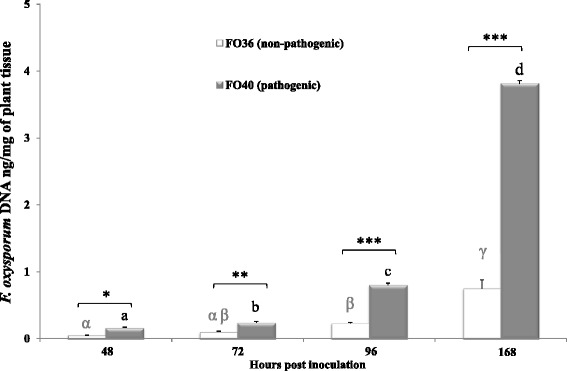
Amount of *F. oxysporum* constitutive gene *translation elongation factor* 1 *alpha* in roots of resistant Forrest soybean genotype inoculated with isolates FO36 (**white boxes**) and FO40 (**grey boxes**) of *F. oxysporum*, over a time course of 168 h. DNA quantities reported as ng of fungal DNA per mg of plant fresh tissue used for DNA extraction. Values represent the mean of three pools of roots for each time-point, where each pool derived from the mixing of five different roots. Same letters over the histograms state not significant differences between means of non-pathogenic (**greek letters**) and pathogenic (**latin letters**) inoculated samples, as resulting from Tukey’s HSD test (P ≤ 0.05). *indicate significant differences between non-pathogenic and pathogenic inoculated means within the same time of sampling, according to two-way ANOVA (*P ≤ 0.05; **P ≤ 0.01; ***P ≤ 0.001)

The highest quantity of fungal DNA was measured at seven days, and five times more fungal DNA (3.81 ng vs. 0.75 ng) was detected for the interaction with the pathogenic isolate (FO40) vs. the non-pathogenic isolate (FO36) (Fig. 1). This profile was indicative of an enhanced reaction of compatibility between the host and the pathogenic isolate, compared to the non-pathogenic one, at the later stages of inoculation and it suggests that the plant may already react to the invading pathogens in the first 96 h. Therefore, 72 and 96 hpi were selected as the crucial time points for RNA-Seq analysis, because they were intended to take into account not only the plant defense responses at the early-intermediate stages of infection, but also to allow the detection of pathogen transcripts when infection was established.

Furthermore, disease severity index (DSI) was evaluated in seedlings at seven days after inoculation. Both isolates were able to infect and colonize soybean seedlings, although to different degrees (2.29 vs. 6.71 mean DSI for the non-pathogenic and pathogenic isolates, respectively; data not shown).

As expected, minor symptoms occurred in plants inoculated with isolate FO36, as also stated by qPCR (Fig. 1). The FO36 isolate produced fewer macroconidia in culture compared to the pathogenic one, which may have contributed to the relatively lower levels of disease and fungal DNA in seedlings over a time course of seven days.

### RNA-Seq analysis of host transcriptome

Root samples were taken from plants, either noninoculated or inoculated with non-pathogenic and pathogenic isolates of *F. oxysporum*, at 72 and 96 hpi. To investigate the comprehensive gene expression profiles of *G. max* and its pathogen *F. oxysporum* simultaneously at the early stages of infection, we analysed transcriptomes obtained from sequencing RNA from eighteen samples, which included three biological replicates for each condition (control/inoculated) and times of inoculation (72 and 96 hpi). A total of about 126, 121 and 140 million 100 bp paired-end reads were generated from noninoculated, FO36 and FO40-inoculated samples, respectively, constituting approximately 12.6, 12.1 and 14 Gb of cDNA sequencing data (Additional file 2: Table S2). On average, 87.2 % of the total reads mapped uniquely to the soybean Williams 82 reference genome (Additional file 2: Table S2). A smaller number of uniquely aligned reads mapped to the *F. oxysporum* reference genome (90,770 in total), and 54,965 aligned reads were from the pathogenic isolate at 96 hpi (Additional file 2: Table S2). The fungal mapping results were consistent with the fungal DNA quantities assessed by qPCR, where fungal DNA increased throughout the course of infection, in particular for the pathogenic isolate, confirming the larger amount of reads detected for this isolate in the later inoculation stage (Fig. 1; Additional file 2: Table S2).

Unfortunately, given the very low read counts of *F. oxysporum*, in particular for the non-pathogenic isolate, further downstream analyses were only performed for soybean and not for *F. oxysporum*. The abundance of soybean transcripts are expressed in upper quartile normalized counts as calculated by HTSeq-count and QuasiSeq programs [20, 21]. One of the three biological replicates for the FO36 inoculated sample at 96 hpi was excluded for further expression level analysis due to a relatively small number of reads. We detected in total 44,026 soybean known protein coding expressed genes (Additional file 3: Table S3).

Soybean expression profiles of FO36 and FO40 *F. oxysporum* isolates inoculated roots were compared with their respective noninoculated controls at 72 hpi and 96 hpi. Genes were considered significantly differentially expressed (DE) if the Qvalue cut-off was below 0.05 and highly differentially expressed (HDE) if the absolute fold change (FC) was ≥ 1.9 [21].

**Fig. 2:**
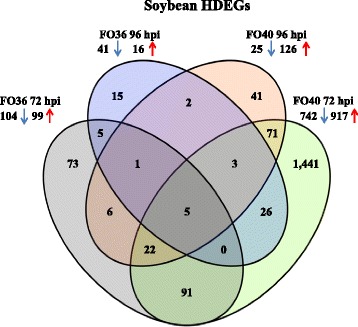
Venn diagram of soybean highly differentially expressed genes (HDEGs) at two inoculations stages (72 and 96 h post inoculation) with *F. oxysporum* FO36 and FO40 isolates, respectively, in the resistant soybean genotype Forrest. Blue coloured down arrow indicates down-regulation of genes and red coloured up arrow indicates up-regulation

RNA-Seq analysis identified 8,471 DEGs. Significant enrichment testing was performed for GO categories and Mercator bins. A Fisher’s exact test was used to determine GO term enrichment (http://www.soybase.org/goslimgraphic_v2/dashboard.php) on DEGs with a Qvalue less than 0.05. Soybean version 2 gene models were further annotated into Mercator functional categories that are easily visualized as MapMan bins and a Wilcox Rank Sum test inside MapMan identified several significantly enriched bins (Additional files 4 and 5: Tables S4 and S5). Due to the high number of DEGs an additional filter of a FC greater than 1.9 was applied for downstream analysis and visualization. This filter resulted in 1,802 soybean HDEGs (Fig. 2; Additional file 6: Table S6). These HDEGs were annotated using Blast2GO software [22] to classified in 13 broader functional categories not provided in the GO terms downloaded from Soybase.org.

### Identification and functional categorization of *F. oxysporum*-responsive genes

A numerical overview of the differences in highly differentially expressed soybean genes at each time point and in each interaction is provided in Fig. 2. We identified 203 and 57 HDEGs in response to the non-pathogenic isolate, and 1,659 and 151 HDEGs in response to the pathogenic isolate at 72 and 96 hpi, respectively. A higher number of HDEGs was observed at 72 hpi compared to 96 hpi and this number was always higher in response to the pathogenic isolate at both times of inoculations. Some overlap in the HDEGs identified between the isolates was observed at 72 hpi, where more than 50 % of HDEGs detected in response to the non-pathogenic isolate was in common with the pathogenic one; however larger changes in expression levels were specifically identified in the interaction with the pathogenic isolate (1,441 HDEGs; Fig. 2). More limited transcriptional changes were found at 96 hpi, although the host responses against the pathogenic isolate continued to be numerically higher.

**Fig. 3:**
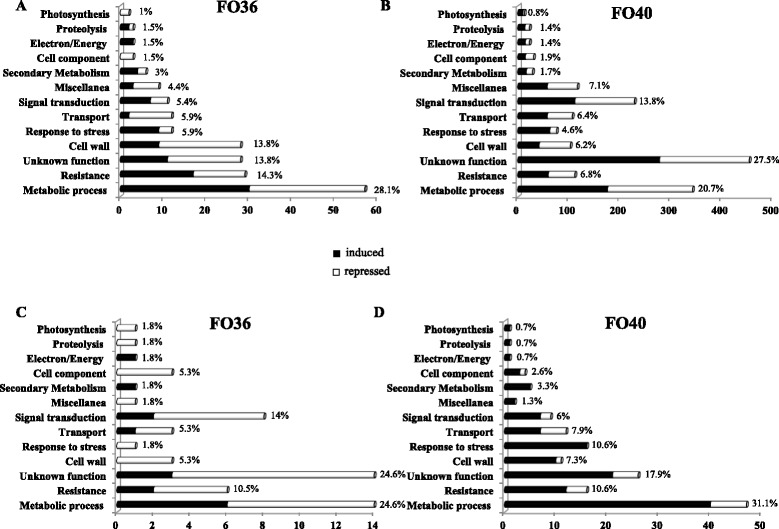
Functional categories of highly differentially expressed genes (HDEGs) in Forrest genotype after *F. oxysporum* FO36 and FO40 isolate inoculation at 72 (**a**, **b**) and 96 (**c**, **d**) hours post inoculation (hpi). HDEGs were annotated by Blast2GO analysis and classified in functional categories on the basis of literature evaluation. Induced genes are represented in black, while repressed ones are in white. The total percentage of modulated transcripts within each functional category is also shown. The complete list of genes is available in Additional file 6: Table S6

In order to understand the biological processes associated with host reactions to *F. oxysporum* infection, soybean HDEGs were assigned to different functional categories (Fig. 3). After discarding genes with unassigned function, in both interactions the largest proportion of HDEGs belonged to metabolic process and defense-related functional classes, such as resistance, response to stress, cell wall and secondary metabolism. In the interaction with the non-pathogenic isolate, metabolic process and defense-related functions accounted for 28.1 and 37 % of the modulated genes at 72 hpi, falling to 24.6 and 19.4 % at 96 hpi, respectively (Fig. 3a and c). An opposite trend was observed in response to the pathogenic isolate and the two functional classes accounted for about 19–21 % at 72 hpi, increasing to 31–32 % in the later stage of inoculation (Fig. 3b and d). Moreover, while at 72 hpi a similar percentage of up and down-regulated genes within each category was modulated in either of the interactions (Fig. 3a and b), at 96 hpi the majority of HDEGs was down-regulated in response to the non-pathogenic isolate, and up-regulated in response to the pathogenic one (Fig. 3c and d). These results suggest markedly different molecular events occurred in response to the two isolates. Even though soybean also activated a certain number of metabolic and defense events against the non-pathogenic isolate in the earlier phases of root colonization, the small number of modulated genes identified at 96 hpi, 72 % of which down-regulated, confirms the lower efficiency in infection and spread of the non-pathogenic isolate.

**Fig. 4:**
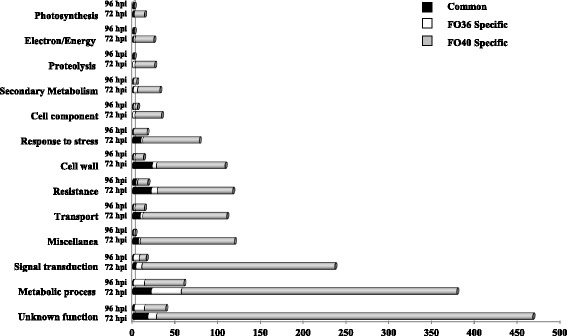
Specificity of transcriptional changes within each functional category in inoculated partially resistant soybean genotype Forrest. Proportion of genes specifically modulated in response to non-pathogenic *F. oxysporum* isolate FO36 (white) and pathogenic isolate FO40 (light grey) or common in response to both isolates (black) at 72 and 96 h post inoculation (hpi)

Figure 4 reports the proportion of genes whose induction/repression in response to inoculation was observed in both interactions or was restricted to one or the other at 72 and 96 hpi. The data show clearly that most of the transcriptional modulation observed in response to the pathogenic isolate had no parallel for the non-pathogenic one, confirming that many of the changes in all functional categories were restricted to FO40 isolate. In contrast, most of the transcriptional modulation observed in response to FO36 also occurred in response to FO40 (Fig. 4).

**Fig. 5:**
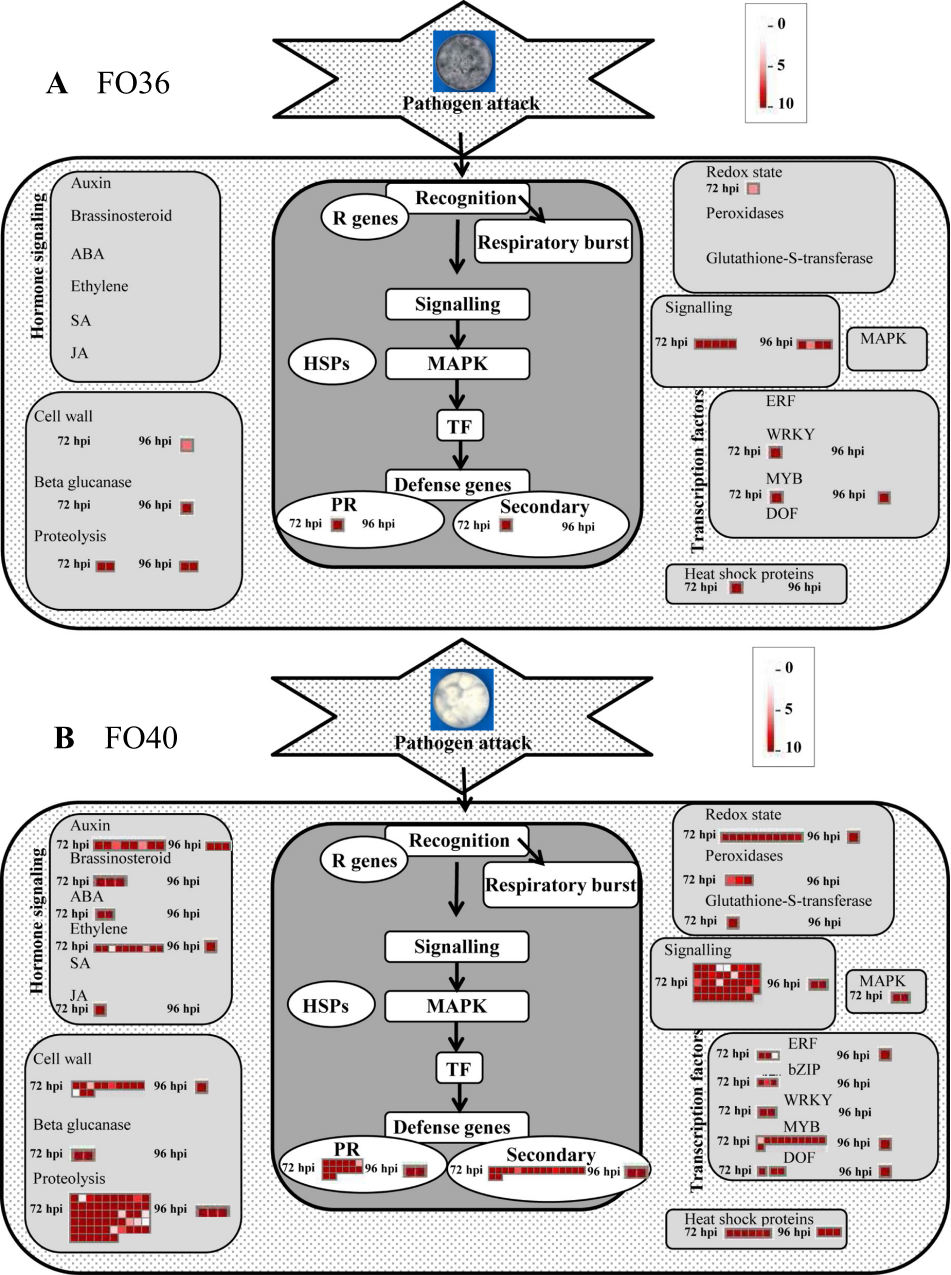
Distribution of specific highly differentially expressed soybean genes in response to non-pathogenic *F. oxysporum* isolate FO36 (**a**) and pathogenic isolate FO40 (**b**), related to biotic stress processes, visualized by MapMan. Each square represents the normalized count value for a single gene in soybean inoculated roots at 72 and 96 h post inoculation (heatmap on the left and on the right within each category, respectively)

By visualizing the specific transcriptional changes related to biotic stress processes at 72 and 96 hpi as annotated by MapMan software [23], as expected, different patterns were observed in response to the non-pathogenic and pathogenic isolates (Fig. 5a and b). HDE soybean genes were well represented for all pathways in response to FO40 isolate, but not for the response to FO36 (Fig. 5a and b). The major differences in host response to infection by the two fungi were in genes with hormone signalling-associated activities, peroxidases, glutathione-S-transferases and mitogen-activated protein kinases, which were totally underrepresented after FO36 inoculation (Fig. 5a). These observations strongly suggest that the limited number of genes specifically modulated in response to the non-pathogenic isolate were not particularly informative and on the whole it appeared that soybean mounted a much less specific response to FO36 inoculation, which may be considered as an unsuccessful attempt to colonize the root tissue.

Consistent with these findings, we focused on soybean HDEGs mainly induced in response to the non-pathogenic isolate or to the pathogenic one, considering the following filters: number of normalized counts (NC) ≤0 and 0.5 in the control sample and NC ≥5 in the inoculated sample. A higher number of HDEGs was detected in response to the pathogenic isolate at both sampling times (11 vs. 48 HDEGs in total in response to the FO36 and FO40 isolates, respectively), as well as higher expression values (Additional files 6 and 7: Tables S6 and 7). These expression values ranged from 5 to 69.7 NC for the non-pathogenic isolate, and from 5 to 224.7 NC for the pathogenic one, at 72 hpi. At 96 hpi, only five genes were induced in response to FO40 isolate, with the highest expression value of 83.3 NC, while no genes were found in response to the non-pathogenic isolate. Overall, these data show that HDEGs belonging to all functional classes, in particular for the metabolic process and defense-related categories, were mainly overexpressed in the interaction with FO40 isolate, confirming a greater stimulation of soybean defense responses to the pathogenic inoculation.

**Fig. 6:**
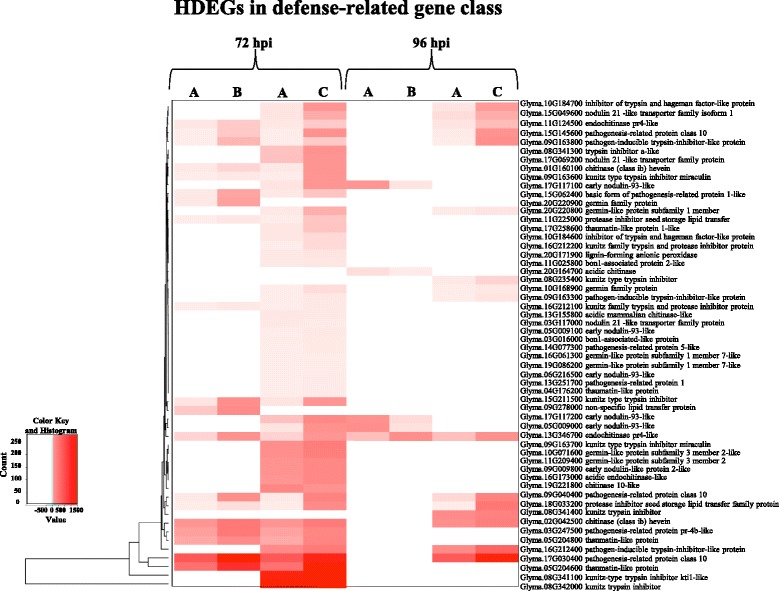
Clustering and heatmap of soybean highly differentially expressed genes (HDEGs) in the defense-related gene class. Description of each gene as obtained from Blast2GO annotation are shown. Light and dark red indicate lower and higher expression values, respectively. White indicates genes that are not DE. Expression heatmaps were plotted using normalized read counts. **a**: Control; **b**: inoculated with non-pathogenic *F. oxysporum* isolate FO36; **c**: inoculated with pathogenic *F. oxysporum* isolate FO40

An example of the expression patterns for HDEGs associated with defense mechanisms against invading pathogens is depicted by a heatmap in Fig. 6. We identified fifty-six genes annotated as defense-related genes, including pathogenensis-related (PR) and thaumatin-like proteins, germins, trypsin and protease inhibitor proteins, and numerous genes related to fungal cell-wall degradation (Fig. 6; Additional files 6 and 8: Tables S6 and 8). In general, the expression of these genes increased significantly at 72 hpi in response to both isolates, but in particular in response to the pathogenic isolate. Later in infection, some of these genes continued to be expressed after FO40 inoculation, while the expression of most of genes returned to control levels (no differential gene expression) for the non-pathogenic interaction. The profiles of these defense-related genes are in line with the general finding that differential gene expression was mainly specific and more pronounced towards the pathogenic isolate.

### Validation of representative genes by real-time RT-PCR

To validate the RNA-Seq expression profiles of soybean HDEGs, the expression levels of several known responsive genes were measured by real-time RT-PCR using gene-specific primers (Additional file 9: Table S9; Additional files 10: Figure S1). Eighteen soybean modulated genes were chosen among those expressed *in planta*, with a preference for defense-related genes, and some of them are marked with a star symbol in the Additional file 8: Table S8, which lists the selected set of *F. oxysporum*-responsive genes. The RT-PCR expression profiles of seventeen soybean genes agreed strongly with the RNA-Seq data, whereas the myb84 gene appeared to be slightly up-regulated according to RT-PCR analysis and slightly reduced from RNA-Seq results at 96 hpi in response to FO36 isolate (Additional file 10: Figure S1).

Overall, the enhancement in the expression levels and defense response revealed by RNA-Seq results in response to the pathogenic isolate were largely concordant with RT-PCR, confirming the reliability of the results.

## Discussion

In order to increase the understanding of transcriptomic responses produced by infection with one of the most important soybean pathogens, *F. oxysporum*, we performed RNA-Seq analysis to compare the global gene expression patterns in soybean roots inoculated with pathogenic and non-pathogenic isolates. The RNA-Seq method is particularly robust for gene expression studies and allows the identification of plant-targeted control strategies, as well as the detection of pathogenicity and virulence factors.

The timing of recognition for *F. oxysporum* infection on soybean is currently unknown, which makes it difficult to propose suitable time points for molecular analysis. We therefore began this investigation by characterizing the infection process in soybean roots inoculated with FO36 and FO40 isolates, in order to determine the optimal sampling times after inoculation. Quantification of fungal growth and disease severity evaluation reported more fungal DNA and elevated symptoms at 168 hpi, and as expected, in particular in response to the pathogenic isolate. As a consequence, this time-point was excluded from the further RNA-Seq analysis, because considered as a too late sample timing. Therefore, 72 and 96 hpi were selected to investigate early-intermediate transcriptional changes, in order to elucidate host genetic responses.

Our study provides the first large-scale investigation of gene expression changes that occur when soybean is inoculated with *F. oxysporum*, and is the first to compare pathogenic and non-pathogenic interactions in the same genetic background, using the partially resistant Forrest genotype. We have identified 205 soybean genes that are highly differentially expressed in pathogenic and non-pathogenic isolates versus control that encode for enzymes that are well known to be involved in defense-related networks. Of these genes, 90 were found to be significantly enriched in GO categories as determined by a Fisher’s exact or in MapMan bin categories by a Wilcox Rank Sum test (Additional files 5 and 8: Tables S5 and S8). Although the remaining 114 genes were not enriched into one of the defined categories or bins, their known relevance in the defense response literature warrants their further investigation. We placed these genes into four broad categories: signalling-related, defense response, transcriptional regulation, and modulation of secondary and sugar metabolism-related and explored their expression below.

### Expression of signalling-related genes

Plants are able to recognize potential microbial pathogens through pathogen-associated molecular patterns (PAMPs) by host sensors, known as pattern-recognition receptors (PRRs) that initiate a series of defense responses called PAMP-triggered immunity (PTI). Most of these plant receptors belong to family of receptor-like kinases (RLK) [24, 25]. In our study five leucine-rich repeat (LRR) type-RLK were identified at 72 hpi in response to the FO40 isolate, four of them up-regulated. Additionally, several LRR-serine threonine protein kinase (STPK) were specifically induced after pathogenic isolate inoculation at the same time point, except one LRR-STPK (Glyma.16G169900) found down-regulated with both pathogens, and a second one (Glyma.01G004800) specifically induced in response to the non-pathogenic isolate.

Brassinosteroid insensitive 1-associated receptor kinase 1 (BAK1) is another significant PRR in plants, required for PTI triggered by bacterial flagellin and elongation factor Tu. Flagellin-sensitive 2 (FLS2) and BAK1 form a complex to initiate innate immunity in plant [26, 27]. Our results revealed the presence of two BAK1 and one FLS2 genes induced only after pathogenic isolate attack in the early stage of inoculation. As previously reported in a different pathosystem [28, 29], BAK1 played a role in the resistance response of banana roots against to *F. oxysporum* f. sp. *cubense*. Our findings are consistent with the hypothesis that BAK1 also controls plant programmed cell death and immunity to necrotrophic fungi, and bak1 mutants of *Arabidopsis* showed extreme susceptibility to necrotrophic fungi [30]. These results suggest that different surveillance systems exist in response to the pathogenic and non-pathogenic isolates, and only in FO40-inoculated soybean roots the increasing BAK1 and FLS2 levels might potentiate the innate immune response.

Pathogen recognition triggers the activation of downstream signalling networks mediated by protein kinases, in particular mitogen-activated protein kinases (MAPK) [31–33]. Several MAPK and MAPK kinases (MAPKK) genes have been characterized in the response of plants to fungal infection [34]. Consistent with this widely accepted model, we found three MAPKK kinases and two SNF1-related protein kinases specifically induced in response to the pathogenic isolate at 72 hpi.

#### Changes in defense response genes

One of the features of plant defense response is production of PR proteins that have been widely characterized and classified into the PR1-17 families [35]. In our research, a high number of genes representing most of 17 different PR were clearly identified as induced after inoculation mainly in the early time point often shared by both isolates, such as three PR10, one PR4, one PR1 and four chitinases (family PR3). Other PR genes up-regulated only in response to the FO40 isolate at 72 hpi included: thaumatin-like proteins (family PR5; four genes), endoglucanases (family PR2; four genes) and lignin forming peroxidase (family PR9; one gene). Furthermore, the same three PR10 proteins and three out of four chitinases induced by both pathogens at 72 hpi, were also specifically up-regulated at 96 hpi in response to the pathogenic isolate but not by FO36. The same trend was also observed for two germin proteins (Glyma.10G168900; Glyma.20G220800), which are known to function in ROS production and biotic and abiotic stress responses [36]. Two additional FO40-induced genes encoding bon1-associated proteins (BAP) were up-regulated at 72 hpi in FO40-inoculated roots. BAP1 and BAP2 are homologous proteins containing a calcium-dependent phospholipid-binding C2 domain and both function in the defense pathway [37]. Furthermore, several nodulin proteins were strongly induced by FO40 at 72 hpi. Interestingly, one of them continued to be induced at 96 hpi for the pathogenic isolate (Glyma.15G049600), whereas three of them were down-regulated after non-pathogenic inoculation, suggesting a potential role in plant defense [19, 38, 39].

In our analysis another prominent class of defense-related genes was represented by protease inhibitors. In total, we identified eighteen genes up-regulated by *F. oxysporum* inoculation, encoding various types of protease inhibitors. Remarkably, 16 and seven out of them were strongly induced (up to 17,701 NC; Glyma.08G342000) only in response to the pathogenic isolate at 72 and 96 hpi, respectively. Indeed, fewer protease inhibitors genes, six and two, were induced by both isolates at the early and later stages of inoculation, respectively. This observation is in line with previous reports on the important role of protease inhibitors in the defense against pathogens [40].

Overall, the induction and greater accumulation of PR, cell wall-degrading enzymes and proteinase inhibitor genes suggested that the degradation of cell wall components of pathogens and proteolysis inhibition are important defense reactions in soybean against *F. oxysporum* isolates at the early infection stage (72 hpi). It could be speculated that the defense responses initiated at the early stage of fungal pathogen infection continued to play a role at the later stage following infection by isolate FO40, while these responses dropped off after 72 hpi for the non-pathogenic isolate.

#### Genes involved in transcriptional regulation

Transcriptome profiling analysis identified a large number of genes encoding TFs, such as ethylene-responsive transcription factors (ERF), MYB, bHLH, GATA, PLATZ and WRKY, and found that their expression was significantly changed upon FO40 inoculation. The ERF TFs play crucial roles in regulating plant responses to environmental stresses [41] and their overexpression in transgenic plants can confer enhanced disease resistance against different types of pathogens [42, 43]. The ERF TFs are known to be involved in ethylene signalling [43], and in agreement with this we also found that the pathogenic isolate infection up-regulated five genes for 1-aminocyclopropane-1-carboxylate (ACC) oxidases at 72 hpi. These enzymes participate in the last step of biosynthesis of ethylene, which has been associated with both wilting and resistance against vascular diseases [44, 45]. The induction of ACC oxidases only by the pathogenic isolate, suggests that ethylene might be involved in resistance against FO40 isolate.

Other strongly represented TF families were *N*AM-*A*TAF1/2-*C*UC2 (NAC) TFs. *Arabidopsis* NAC may play a dual role in regulating both jasmonic acid (JA)- and abscisic acid (ABA)-dependent responses and manipulate plant stress responses by activating other genes encoding MYB TFs, amylase, cold responsive protein, dehydration responsive proteins, glutathione-S-transferases, and late embryogenesis abundant (LEA) proteins [46, 47]. We observed that the percentage of up-regulated NAC and MYB TFs was between 60–70 % of total at 72 hpi for the pathogenic isolate, while no NAC and one down-regulated MYB gene were observed in response to FO36 isolate. Accordingly, two LEA genes were specifically induced by the pathogenic isolate at 72 hpi. *Arabidopsis* R2R3-MYB TF directly acts on the promoters of the flavonoid biosynthesis genes and in the signalling chain that causes flavonol-specific gene activation in phenylpropanoid biosynthesis [48]. In line with this, we also observed differential expression of genes encoding enzymes related to biosynthesis of flavonol and phenylpropanoid.

#### Modulation of secondary and sugar metabolism-related genes

As previously stated, our results demonstrated the transcriptional induction of multiple genes involved not only in the phenylpropanoid pathway itself, but also in the downstream flavonoid and diterpenoid biosynthesis pathways. More specifically, phenylpropanoid-related genes, such as phenylalanine ammonia lyase (PAL), 4-coumarate ligase (4CL), trans-cinnamate 4-monooxygenase (C4M) and caffeoyl-o-methyltransferase (COMT) were consistently modulated upon pathogenic isolate inoculation mainly at the early time point. RNA-Seq data also revealed significant changes in genes belonging to the flavonoid pathway upon inoculation, especially with the FO40 isolate, such as chalcone isomerase (CI), flavonoid 3-hydroxylase (F3H) and flavonol synthase (FS), as well as two terpene synthases (TPs) that were identified. This finding once again indicated that lignification and cell wall strengthening are active resistance mechanisms in the soybean-pathogen interaction, as already observed in other pathosystems [49–52], as well as flavonoids and isoflavonoids, which could be crucial metabolites for the soybean infection response [53, 54].

Activation of defense responses upon pathogen infection is usually accompanied by a rapid induction of sink metabolism, possibly to satisfy the increased demand for carbohydrates as an energy source to sustain the cascade of cost-intensive direct defense responses and further mediate physiological adaptations [55]. Additionally, pathogens try to manipulate plant carbohydrate metabolism for their own needs [56, 57]. In our study, we identified eight up-regulated genes belonging to the carbohydrate metabolic process group, seven out of them specifically induced after pathogenic inoculation at 72 hpi, and one cell wall invertase (CWI) (Glyma.15G024600) induced by the same isolate at both time points. Enhanced expression and activity of CWIs has been reported in several plant-pathogen interactions and is essential to modulate sugar partitioning necessary for the pathogen development [58, 59]. Moreover, during infection CWI activity also triggers plant defense responses, such as induction of PR genes, PAL gene expression, callose deposition and reduction of cell death, in agreement with our previous finding. One of the other possibilities for changing the sugar content is the regulation of the expression of the sugar transporters. We observed the up-regulation of five bidirectional sugar transporters only in response to the FO40 isolate at 72 hpi. In rice infected by *Xanthomonas oryzae* pv. *Oryzae*, SWEET proteins were up-regulated and sucrose accumulated in apoplast ready to be used for the pathogen growth [60, 61]. Santi and co-workers [62, 63] reported that sucrose transporter genes are first down-regulated during infection of grapevine by stolbur phytoplasma to limit the spread and then up-regulated during the recovery stage providing necessary nutrients [62, 63]. Together, these results substantiate the active mobilization of sucrose in FO40-inoculated soybean roots, providing ready-to-use sugars for pathogen growth and plant immunity.

## Conclusions

Using RNA-Seq, we analysed the expression profiles of soybean roots inoculated with non-pathogenic/pathogenic fungal isolates at 72 and 96 hpi. We identified markedly differential responsive expression patterns in the two host-pathogen combinations and more active and drastic reactions were observed in response to the pathogenic isolate. This response included a stronger activation of many well-known defense-related genes, and several genes involved in ethylene biosynthesis and signalling, TFs, secondary and sugar metabolism. The high degree of activation of host defense signalling pathways identified in soybean was consistent with the more extensive necrosis and the highest quantity of fungal DNA observed for the pathogenic isolate. These results may be useful to understand the molecular basis of soybean-*F. oxysporum* interactions, and also in the development of new resistance mechanisms in soybean against *F. oxysporum*, including the over-expression of key soybean genes.

## Methods

### Plant and fungus material

Soybean [*G. max* (L.) Merrill] partially resistant genotype Forrest [7, 64] was evaluated after inoculation with a conidial suspension of non-pathogenic FO36 and pathogenic FO40 *F. oxysporum* isolates. The FO36 and FO40 isolates were collected from Lyon and Butler counties in Iowa, respectively, during a 3-year survey from 2007 to 2009 that assessed *Fusarium* spp. diversity and frequency from soybean roots [3]. Aggressiveness of the two isolates was previously determined using rolled-towel assays [7, 8]. Both isolates used in this study were maintained on silica in the dark at 5 °C at Iowa State University, Seed Science Center in Ames, U.S.A.

### Production of the inoculum and inoculation procedure

Inoculum for both isolates was grown for seven days on potato dextrose agar (PDA) at 25 °C with a 12-h photoperiod. Conidia were collected by rinsing plates with sterile water, scraping the agar surface with a scalpel and filtering the conidial suspension through sterile cloth. Spore suspension was adjusted to a final concentration of 1 × 10^6^ conidia/ml based on microscopic counts using a Bürker chamber. Fifteen seeds of the partially resistant Forrest genotype were placed on a paper towel moistened with sterile distilled water and inoculated by pipette with 100 μl of 1 × 10^6^ conidial suspension of FO36 or FO40 isolates. Another moistened paper towel was placed over the inoculated seeds, rolled up, and placed vertically in a 25-l bucket. An open plastic bag was placed over each towel to avoid cross-contamination between isolates. A black plastic bag was placed over each bucket and they were placed on a bench at room temperature (~22 °C). Noninoculated checks were included to ensure that other seed pathogens were not present. For the detection of *F. oxysporum* by fungal DNA assay, roots were sampled at 48, 72, 96 and 168 hpi to evaluate fungal growth and colonization. For RNA-Seq analysis, roots were collected at 72 and 96 hpi. Noninoculated control roots were sampled at the same times listed above. Three pools of five roots were prepared for each isolate and sampling time. The resulting samples were immediately frozen in liquid nitrogen and stored at −80 °C until biological analysis were carried out.

### Quantitation of fungal DNA and disease severity evaluation

After inoculation, roots at 48, 72, 96 and 168 hpi were evaluated to quantify the growth of *F. oxysporum* by qPCR. DNA was extracted from soybean samples, as well as from FO36 and FO40 *F. oxysporum* isolates. To extract fungal DNA, the edge of a culture of each isolate grown for seven days on PDA plates at 25 °C with a 12-h photoperiod was scraped using a sterile loop. The mycelium and spores were transferred to a 50-ml plastic centrifuge tube (Corning Inc., Tewksbury, MA) containing 25 ml of liquid growth medium (3 g of yeast extract, 3 g of malt extract, 5 g of peptone, 20 g of dextrose, 2 g of NH_4_SO_4_ in 1 l of water). The inoculated medium was incubated at room temperature for 5 days on an Innova 2100 platform shaker (New Brunswick Scientific Co., Inc., Enfield, CT) at 100 rpm. Mycelium was harvested by filtration with sterile Miracloth (EMD Biosciences Inc., La Jolla, CA), frozen, and lyophilized. DNA from both isolates and plant tissues was extracted using a conventional CTAB chloroform: isoamyl alcohol protocol [65]. The quality and quantity of DNA was evaluated by measuring the concentration (ng/μl) and A_260_/A_280_ and A_260_/A_230_ via a NanoDrop 2000 spectrophotometer (Thermo Scientific, Wilmington, DE). The genomic DNA was subsequently used as the template for the fungal DNA quantification using qPCR.

Quantitative PCR was carried out on the CFX-96 instrument (Bio-Rad, Hercules, CA). Each DNA sample was loaded in triplicate in a total reaction volume of 20 μl per sample with each reaction mix containing 2 × SYBR Green Mastermix (Applied Biosystems, Foster City, CA) and 0.4 μM of each primer, using primers targeting the *F. oxysporum tef*1*α* gene (JN222908.1; Additional file 9: Table S9). The following cycling conditions were used: 95 °C for a 10 min denaturation step; followed by 38 cycles of amplification at 95 °C for 15 s and 58° for 1 min; and melting curve analysis of heating to 95 °C, cooling to 60 °C for 1 min, and heating to 95 °C at a rate of 0.5 °C/5 s. DNA quantities are reported as ng of fungal DNA obtained from root tissues and determined based on the equation of the linear regression according to the instrument technical manual (Bio-Rad). Fungal DNA (20 ng) deriving from both *F. oxysporum* isolates was serially diluted [1:1, 1:5, 1:5^2^, 1:5^3^, 1:5^4^, 1:5^5^] in sterile water and 20 ng of each root DNA sample was compared to the dilution standard curve to determine fungal DNA quantity.

Two-factor ANOVA was performed on the observed means of the fungal *tef*1*α* DNA quantity, considering times of sampling (48, 72, 96 and 168 hpi) and treatments (non-pathogenic and pathogenic inoculated samples) as fixed factors to test the significance (P ≤ 0.05) of times of sampling, treatments and their interactions. One-factor ANOVA, followed by Tukey’s HSD test (P ≤ 0.05), was performed on the observed means of the fungal *tef*1*α* DNA quantity within each treatment to set significant differences among times of sampling. Differences between non-pathogenic and pathogenic inoculated means within the same time of sampling were performed using two-way ANOVA and considered to be significant at *P ≤ 0.05; **P ≤ 0.01; ***P ≤ 0.001.

Disease severity evaluation was performed on seedlings collected at 168 hpi. The seedlings were rated by measuring the length of hypocotyl tissue (lesion length in mm) and the total plant length (mm). DSI was calculated by dividing the total plant length and multiplying by 100 [66].

### RNA isolation, library preparation and bioinformatic analysis

Root tissues for RNA isolation for RNA-Seq libraries were dissected from soybean samples inoculated at 72 and 96 hpi with FO36 and FO40 *F. oxysporum* isolates and their respective controls (18 samples overall). Frozen tissues (500 mg) were ground in liquid nitrogen with a mortar and pestle. RNA was isolated using Trizol reagent (Invitrogen, Carlsbad, CA) and then purified with the RNA Clean up protocol (Qiagen, Valencia, CA), according to the manufacturer’s instructions. RNA quality and quantity were determined using a Nanodrop 2000 spectrophotometer (Thermo Scientific), as well as by agarose gel electrophoresis. Furthermore, total RNA samples were assessed for quality using an Agilent® 2100 Bioanalyzer TM (Agilent, Santa Clara, CA). RNA-Seq libraries were prepared by the Iowa State University DNA Facility and sequenced using Illumina HiSeq 2500 sequencer (Illumina Inc., San Diego, CA). 100-bp paired-end reads were generated. Mapping of the RNA-Seq data was performed at the Genome Informatics Facility, Iowa State University. Short read sequences were aligned to the soybean Williams 82 reference genome (Glyma.Wm82.a2.v1 genome assembly 2 annotation version 1; http://www.soybase.org) using GSNAP software [67]. Only uniquely mapped reads were considered for downstream differential expression analysis. Soybean gene models were downloaded from Joint Genome Institute and read counts for each gene model were obtained using the HTSeq program developed by Anders and co-workers [21]. Differential expression analysis was performed using the QuasiSeq package in R [20]. For DE analysis, pathogenic isolate (FO40) was compared against non-pathogenic isolate (FO36) at each time point. Upper quartile normalization was used to normalize the read counts for library size. In QuasiSeq, the QLSpline method was used to account for over-dispersion effects. To account for multiple testing, we estimated the Qvalues for each gene model. To control false discovery rate at 5 %, gene models with Qvalues less than 0.05 were declared DE and gene models with Qvalues less than 0.05 and absolute value of FC ≥ 1.9 were declared to be HDE. For the analysis of fungal genes, the short read sequences were aligned to combined soybean and *F. oxysporum* f. sp. *pisi* HDV247 NRRL 37622 reference genome *F.oxysporum* HDV247 genomes (http://www.broadinstitute.org). Due to the very small number of fungal reads, the further normalized expression analysis of transcripts was not performed for *F. oxysporum*. Soybean GO categories were downloaded from Soybase.org. GO term enrichment (http://www.soybase.org/goslimgraphic_v2/dashboard.php; [68]) was determined on DEGs with a Qvalue less than 0.05. Mercator functional categories that are easily visualized as MapMan bins were annotated for all gene models. A Wilcox Rank Sum test inside MapMan was used to identify several significantly enriched bins (Additional files 4 and 5: Tables S4 and S5). Due to the high number of DEGs an additional filter of a fold change greater than 1.9 was applied for downstream analysis and visualization and we refer to these gene as HDEGs.

Sequences of HDEGs were compared with National Center for Biotechnology Information (NCBI) non redundant (NR) database with a Blast E-value of 10^−3^ and were functionally annotated using Blast2GO [22] assigning a GO term and a metabolic pathway in the Kyoto Encyclopedia of Genes and Genomes (KEGG) to the query sequences. Sequences were classified into 13 functional categories (Cell component; Cell wall; Electron/Energy; Metabolic process; Miscellanea; Photosynthesis; Proteolysis; Response to stress; Resistance; Secondary metabolism; Signal transduction; Transport; Unknown function) based on GO annotation.

### Data deposition

The sequencing data have been submitted to Gene Expression Omnibus (GEO) database under the accession number of GSE66861.

### Real-time RT-PCR validation analysis

Gene expression data from RNA-Seq analysis were validated by real-time RT-PCR. This was performed on selected soybean genes that were up- or down-regulated at 72 and 96 hpi. A 1 μg sample of total RNA after DNAse I treatment (Qiagen) was used for cDNA synthesis following the iScript cDNA synthesis kit protocol (Bio-Rad). Twenty ng of single strand cDNA determined by Nanodrop 2000 spectrophotometer (Thermo Scientific) were used for real-time RT-PCR. Relative quantitative analysis was performed under the following conditions: 95 °C for 3 min and 44 cycles at 95 °C 10 s, 60 °C 25 s. A melting curve analysis, ranging from 60 to 95 °C, was used to identify different amplicons, including non-specific products. Three technical replicates (within each biological replicate) were employed for each tested sample and template-free negative controls. Gene-specific primers were designed within consecutive exons, separated by an intron, using Primer3 software and their sequences are shown in Additional file 9: Table S9. Relative quantification was normalized to the soybean housekeeping control genes *actin* and FC in gene expression was calculated using the 2^-ΔΔCt^ method [69].

## Additional files

Additional file 1: Table S1. Means of the fungal *tef*1*α* DNA quantity and their significances, determined by two-way ANOVA (*P* ≤ 0.05), considering treatment and time of sampling as fixed factors. Means followed by the same letter state not significant differences between times of sampling within each treatment, as resulting by one-way ANOVA (*P* ≤ 0.05) and Tukey’s HSD test (*P* ≤ 0.05). (DOCX 16 kb) Additional file 2: Table S2. Mapping results of RNA-Seq reads. Numbers of preprocessed, mapped and uniquely aligned RNA-Seq reads mapping to the soybean and *F. oxysporum* genomes are reported for each biological replicate for control (CTRL) and FO36 and FO40 *F. oxysporum* inoculated Forrest genotype at 72 and 96 h post inoculation (hpi), respectively. (DOCX 30 kb) Additional file 3: Table S3. Expression levels of soybean predicted genes. Gene expression values are reported for all soybean predicted genes in control and inoculated Forrest genotype with non-pathogenic (FO36) and pathogenic (FO40) isolates of *F. oxysporum* at 72 and 96 h post inoculation (hpi). Genes modulated at the different conditions in response to each isolate and time-point are provided in separate sheets for easier access. (XLSX 15482 kb) Additional file 4: Table S4. Output generated from Mercator sequence annotation webserver (http://www.plabipd.de/portal/web/guest/mercator-sequence-annotation). The file can be used as input into MapMan as a mappings file to explore subsets of *Glycine max* version 2 gene models for significant enrichment in any of the Mercator defined bins. (XLSX 9217 kb) Additional file 5: Table S5. GO and Mercator bin enrichment results for FO36 and FO40 at both times post inoculation (72 and 96 hpi). (XLSX 334 kb) Additional file 6: Table S6. Expression levels and annotation results of soybean highly differentially expressed genes (HDEGs) modulated by inoculation with non-pathogenic (FO36) and pathogenic (FO40) isolates of *F. oxysporum* at 72 and 96 h post inoculation (hpi). HDEGs were identified using QuasiSeq package using a Qvalue <0.05. The fold changes and functional annotations are reported for each gene. HDEGs modulated at the different conditions in response to each isolate and time-point are provided in separate sheets for easier access. (XLSX 3049 kb) Additional file 7: Table S7. Comparison between differentially expressed genes in inoculated Forrest genotype with *F. oxysporum* FO36 and FO40 isolates at 72 and 96 hpi. (DOCX 23 kb) Additional file 8: Table S8. A summary of selected soybean FO-responsive genes. Genes that were identified as enriched in a GO category or MapMan bin are marked with a + or Ψ symbol, respectively. (DOCX 88 kb) Additional file 9: Table S9. Details of the real-time RT-PCR analysis. The file contains: the sequence ID of each gene analysed by real-time RT-PCR and the corresponding primer pairs used for the amplification (FOR = forward primer, REV = reverse primer). (XLS 29 kb) Additional file 10: Figure S1. Comparison of RNA-Seq and real-time RT-PCR analysis of selected soybean genes in response to inoculation with *F. oxysporum*. Expression profiles of (A) carbohydrate kinase (Glyma.12G051800), (B) cell-wall invertase (Glyma.15G024600), (C) bidirectional sugar transporter sweet10-like (Glyma.04G198400), (D) chalcone isomerase (Glyma.20G241700), (E) chitinase (Glyma.01G160100), (F) glutathione s-transferase (Glyma.08G174900), (G) laccase-7-like (Glyma.U027300), (H) myb transcription factor myb84 (Glyma.08G042100), (I) pathogenesis-related protein class 10 (Glyma.15G145600), (J) sucrose synthase (Glyma.19G212800), (K) endoglucanase (Glyma.05G236400), (L) trans-cinnamate 4-monooxygenase (Glyma.20G114200), (M) proline-rich protein (Glyma.15G199700), (N) bidirectional sugar transporter sweet3-like (Glyma.04G238100), (O) 1-aminocyclopropane-1-carboxylate oxidase 1 (Glyma.05G222400), (P) seed linoleate 9 s-lipoxygenase (Glyma.08G189600), (Q) peroxidase (Glyma.09G023000), (R) sulphate transporter (Glyma.15G052000). Dotted lines and histograms represent values expressed as fold change of transcript levels in the inoculated FO36 and FO40 *F. oxysporum* samples with respect to the transcript levels in control samples at 72 and 96 hpi assessed by RNA-Seq and real-time RT-PCR analysis, respectively. The asterisk (*) means that the FC revealed by RNA-Seq method for that gene is included among HDEGs. (PPTX 149 kb)
