# Supplementary material for: Transcriptome profiling of soybean (Glycine max) roots challenged with pathogenic and non-pathogenic isolates of Fusarium oxysporum
Source: BMC Genomics. 2015 Dec 21;16:1089. doi: 10.1186/s12864-015-2318-2 (PMC4687377; doi:10.1186/s12864-015-2318-2)
Supplement: Additional file 1: Table S1. — Means of the fungal tef1α DNA quantity and their significances, determined by two-way ANOVA (P ≤ 0.05), considering treatment and time of sampling as fixed factors. Means followed by the same letter state not significant differences between times of sampling within each treatment, as resulting by one-way ANOVA (P ≤ 0.05) and Tukey’s HSD test (P ≤ 0.05). (DOCX 16 kb) [file 12864_2015_2318_MOESM1_ESM.docx]

**Additional file 1: Table S1.** Means of the fungal *tef*1*α* DNA quantity and their significances, determined by two-way ANOVA (*P*≤0.05), considering treatment and time of sampling as fixed factors. Means followed by the same letter state not significant differences between times of sampling within each treatment, as resulting by one-way ANOVA (*P*≤0.05) and Tukey’s HSD test (*P*≤0.05).

***=significance at 0.001

| **Source** | ***tef*1*α*** |
| --- | --- |
| **Hours post inoculation (hpi)** | *** |
| 48 | 0.10650 |
| 72 | 0.17717 |
| 96 | 0.53450 |
| 168 | 2.28167 |
| **Treatment** | *** |
| Non-pathogenic (FO36) | 0,28683 |
| Pathogenic (FO40) | 1.26308 |
| **hpi X Treatment** | *** |
| **Overall average** | **0.77496** |
| **Among non-pathogenic inoculated samples**  **(Tukey’s HSD)** | |
| 48 | 0.05000 α |
| 72 | 0.10967 αβ |
| 96 | 0.23433β |
| 168 | 0.75333 γ |
| **Among pathogenic inoculated samples**  **(Tukey’s HSD)** | |
| 48 | 0.16300 a |
| 72 | 0.24467 b |
| 96 | 0.83467 c |
| 168 | 3.81000 d |
