# Supplementary material for: Transcriptome profiling of soybean (Glycine max) roots challenged with pathogenic and non-pathogenic isolates of Fusarium oxysporum
Source: BMC Genomics. 2015 Dec 21;16:1089. doi: 10.1186/s12864-015-2318-2 (PMC4687377; doi:10.1186/s12864-015-2318-2)
Supplement: Additional file 2: Table S2. — Mapping results of RNA-Seq reads. Numbers of preprocessed, mapped and uniquely aligned RNA-Seq reads mapping to the soybean and F. oxysporum genomes are reported for each biological replicate for control (CTRL) and FO36 and FO40 F. oxysporum inoculated Forrest genotype at 72 and 96 h post inoculation (hpi), respectively. (DOCX 30 kb) [file 12864_2015_2318_MOESM2_ESM.docx]

**Additional file 2: Table S2.** Mapping results of RNA-Seq reads. Numbers of preprocessed, mapped and uniquely aligned RNA-Seq reads mapping to the soybean and *Fusarium oxysporum* genomes are reported for each biological replicate for control (CTRL) and FO36 and FO40 *F. oxysporum* inoculated Forrest genotype at 72 and 96 hours post inoculation (hpi), respectively.

| Samples | | N^o^. of preprocessed reads | N^o^. of plant total mapped reads^*^ | N^o^. of plant uniquely aligned mapped reads^*^ | N^o^. of fungal total mapped reads^†^ | N^o^. of fungal uniquely aligned mapped reads^†^ |
| --- | --- | --- | --- | --- | --- | --- |
| ^a^CTRL | **72** ^d^**hpi** **^e^R1** | 21,720,710 | 21,034,162 | 20,480,474 | **/** | **/** |
|  | **72 hpi** **R2** | 22,579,456 | 13,940,858 | 12,079,682 | **/** | **/** |
|  | **72 hpi** **R3** | 18,479,603 | 17,977,673 | 17,238,502 | **/** | **/** |
|  | **96 hpi R1** | 25,201,068 | 22,927,819 | 22,101,224 | **/** | **/** |
|  | **96 hpi R2** | 18,284,685 | 17,921,173 | 17,066,300 | **/** | **/** |
|  | **96 hpi R3** | 19,525,107 | 19,127,069 | 18,010,722 | **/** | **/** |
| ^b^FO36 | **72 hpi R1** | 24,301,369 | 23,415,371 | 22,522,633 | 4,306 | 4,259 |
|  | **72 hpi** **R2** | 25,872,294 | 24,976,323 | 23,553,305 | 3,550 | 3,512 |
|  | **72 hpi** **R3** | 18,118,275 | 17,634,639 | 16,785,183 | 2,292 | 2,272 |
|  | **96 hpi R1** | 23,859,446 | 23,464,105 | 22,212,322 | 1,764 | 1,738 |
|  | **96 hpi R2** | 24,753,279 | 23,279,983 | 22,262,682 | 2,195 | 2,158 |
|  | **96 hpi R3** | 3,904,571 | 142,956 | 125,319 | 3,081 | 3,014 |
| ^c^FO40 | **72 hpi R1** | 24,921,639 | 24,519,704 | 23,189,082 | 8,877 | 8,831 |
|  | **72 hpi** **R2** | 20,426,119 | 19,577,382 | 18,775,468 | 6,771 | 6,740 |
|  | **72 hpi** **R3** | 22,941,356 | 20,056,758 | 18,739,460 | 9,401 | 9,347 |
|  | **96 hpi R1** | 23,643,663 | 22,502,147 | 21,326,764 | 28,998 | 28,881 |
|  | **96 hpi R2** | 23,312,628 | 22,454,039 | 21,349,023 | 18,741 | 18,659 |
|  | **96 hpi R3** | 24,461,071 | 20,706,144 | 18,987,567 | 7,485 | 7,425 |

^a^CTRL= Control, uninoculated; ^b^FO36= non-pathogenic *Fusarium oxysporum* isolate; ^c^FO40= pathogenic *F. oxysporum* isolate; ^d^hpi= hours post inoculation; ^e^R=biological replication.

^*^Mapped on the soybean Williams 82 reference genome (Glyma.Wm82.a2.v1 genome assembly 2 annotation version 1; http://www.soybase.org).

^†^Mapped on the fungus *F. oxysporum* f. sp. *pisi* HDV247 NRRL 37622 reference genome (http://www.broadinstitute.org).
