# Supplementary material for: Transcriptome profiling of soybean (Glycine max) roots challenged with pathogenic and non-pathogenic isolates of Fusarium oxysporum
Source: BMC Genomics. 2015 Dec 21;16:1089. doi: 10.1186/s12864-015-2318-2 (PMC4687377; doi:10.1186/s12864-015-2318-2)
Supplement: Additional file 7: Table S7. — Comparison between differentially expressed genes in inoculated Forrest genotype with F. oxysporum FO36 and FO40 isolates at 72 and 96 hpi. (DOCX 23 kb) [file 12864_2015_2318_MOESM7_ESM.docx]

**Additional file 7: Table S7.** Comparison between highly differentially expressed genes in inoculated Forrest genotype with *Fusarium oxysporum* FO36 and FO40 isolates at 72 and 96 hpi.

| Functional category | N° of genes | | | |
| --- | --- | --- | --- | --- |
|  | **0≤ NC* CTRL ≤0.5; NC INOC. ≥5** | | | |
|  | **72 hpi FO36** | **72 hpi FO40** | **96 hpi FO36** | **96 hpi FO40** |
| Total | 11 | 43 | / | 5 |
| Defense-related genes | 6 | 12 | / | 1 |
| *Cell wall* | 2 | 3 | / | / |
| *Resistance* | 1 | 3 | / | 1 |
| *Response to stress* | 3 | 6 | / | / |
| Cell component | / | 1 | / | / |
| Metabolic process | 5 | 14 | / | 2 |
| Miscellanea | 1 | 3 | / | / |
| Signal transduction | / | 1 | / | 1 |
| Transport | / | 2 | / | / |
| Unknown function | / | 11 | / | 1 |

*** NC=Normalized counts**

The table shows the total number of genes whose induction is higher in response to pathogenic FO40 or non-pathogenic isolate FO36 isolate within each category for each time of inoculation, considering the following filters: 0≤ NC* CTRL ≤0.5; NC INOC. ≥5. Defense-related genes considered for the comparison are those functionally categorized as ‘cell wall’, ‘resistance’, ‘response to stress’ and ‘secondary metabolism’.
