# Supplementary material for: Transcriptome profiling of soybean (Glycine max) roots challenged with pathogenic and non-pathogenic isolates of Fusarium oxysporum
Source: BMC Genomics. 2015 Dec 21;16:1089. doi: 10.1186/s12864-015-2318-2 (PMC4687377; doi:10.1186/s12864-015-2318-2)
Supplement: Additional file 8: Table S8. — A summary of selected soybean FO-responsive genes. Genes that were identified as enriched in a GO category or MapMan bin are marked with a + or Ψ symbol, respectively. (DOCX 88 kb) [file 12864_2015_2318_MOESM8_ESM.docx]

| Gene ID  **Additional file 8: Table S8**. A summary of selected soybean FO-responsive soybean genes. | FO36 NC | FO40 NC | Up/Down | FO36 NC | FO40 NC | Up/Down | Annotation | |
| --- | --- | --- | --- | --- | --- | --- | --- | --- |
|  | 72 hpi | | | 96 hpi | | |  |  |
| Kinases | | | | | | | |  |
| Glyma.15G214300 | /^†^ | 2 | ↑ | / | / |  | cysteine-rich rlk (receptor-like kinase) protein | |
| Glyma.13G266000 | / | 15 | ↑ | / | / |  | lrr receptor-like kinase | |
| Glyma.18G254400Ψ | / | 23 | ↑ | / | / |  | lrr receptor-like kinase family protein | |
| Glyma.18G254300Ψ | / | 66 | ↑ | / | / |  | lrr receptor-like kinase family protein | |
| Glyma.16G065200Ψ | / | 10 | ↓ | / | / |  | lrr receptor-like kinase family protein | |
| Glyma.01G152200Ψ | / | 28 | ↑ | / | / |  | lrr receptor-like kinase family protein | |
| Glyma.13G003500Ψ | / | 8 | ↑ | / | / |  | lrr receptor-like serine threonine-protein kinase fls2-like | |
| Glyma.16G172200Ψ | / | 4 | ↑ | / | / |  | lrr receptor-like serine threonine-protein kinase gso2-like | |
| Glyma.16G169900Ψ | 4 | 13 | ↓; ↓ | / | / |  | lrr receptor-like serine threonine-protein kinase gso2-like | |
| Glyma.13G268100Ψ | / | 43 | ↑ | / | / |  | probable lrr receptor-like serine threonine-protein kinase | |
| Glyma.18G250500Ψ | / | 35 | ↑ | / | / |  | probable lrr receptor-like serine threonine-protein kinase | |
| Glyma.11G073800 | / | 157 | ↑ | / | / |  | probable lrr receptor-like serine threonine-protein kinase | |
| Glyma.15G084500 | / | 48 | ↑ | / | / |  | probable lrr receptor-like serine threonine-protein kinase | |
| Glyma.16G092600 | / | 360 | ↑ | / | / |  | probable lrr receptor-like serine threonine-protein kinase | |
| Glyma.01G004800 + | 51 | / | ↑ | / | / |  | probable lrr receptor-like serine threonine-protein kinase | |
| Glyma.05G119600+ | / | 84 | ↑ | / | / |  | brassinosteroid insensitive 1-associated receptor kinase 1-like | |
| Glyma.07G143900 | / | 94 | ↑ | / | / |  | brassinosteroid lrr receptor kinase-like isoform x1 | |
| Glyma.15G048500+Ψ | / | 37 | ↑ | / | / |  | mitogen-activated protein kinase kinase kinase 1-like | |
| Glyma.14G195300 | / | 30 | ↑ | / | / |  | mitogen-activated protein kinase kinase kinase npk1-like | |
| Glyma.17G245300 | / | 16 | ↑ | / | / |  | mitogen-activated protein kinase kinase kinase yoda-like isoform x1 | |
| Glyma.06G170700 | / | 228 | ↑ | / | / |  | snf1-related protein kinase regulatory subunit gamma-1-like | |
| Glyma.04G195300 | / | 116 | ↑ | / | / |  | snf1-related protein kinase regulatory subunit gamma-1-like | |
| Pathogenesis-related and other known defense-related genes | | | | | | | |  |
| Glyma.15G145600* | 63 | 171 | ↑; ↑ | / | 155 | ↑ | pathogenesis-related protein class 10 | |
| Glyma.09G040400 | 206 | 363 | ↑; ↑ | / | 349 | ↑ | pathogenesis-related protein class 10 | |
| Glyma.17G030400 | 2,537 | 1,716 | ↑; ↑ | / | 1877 | ↑ | pathogenesis-related protein class 10 | |
| Glyma.03G247500+ | 477 | 308 | ↑; ↑ | / | / |  | pathogenesis-related protein pr-4b-like | |
| Glyma.15G062400Ψ | 90 | 67 | ↑; ↑ | / | / |  | basic form of pathogenesis-related protein 1-like | |
| Glyma.13G251700Ψ | / | 4 | ↑ | / | / |  | pathogenesis-related protein 1 | |
| Glyma.14G077300Ψ | / | 7 | ↑ | / | / |  | pathogenesis-related protein 5-like | |
| Glyma.01G160100*+ | 56 | 127 | ↑; ↑ | / | / |  | chitinase (class ib) hevein | |
| Glyma.02G042500 + | 480 | 434 | ↑; ↑ | / | 403 | ↑ | chitinase (class ib) hevein | |
| Glyma.13G346700+ | 165 | 114 | ↑; ↑ | 187 | 158 | ↑; ↑ | endochitinase pr4-like | |
| Glyma.11G124500+ | 63 | 63 | ↑; ↑ | / | 74 | ↑ | endochitinase pr4-like | |
| Glyma.16G173000+Ψ | / | 287 | ↑ | / | / |  | acidic endochitinase-like | |
| Glyma.19G221800+ | / | 146 | ↓ | / | / |  | chitinase 10-like | |
| Glyma.20G164700+Ψ | / | / |  | 4 | ↓ |  | acidic chitinase | |
| Glyma.13G155800+ | / | 8 | ↓ | / | / |  | acidic mammalian chitinase-like | |
| Glyma.05G204600+ | / | 2,909 | ↑ | / | / |  | thaumatin-like protein | |
| Glyma.05G204800+ | / | 314 | ↑ | / | / |  | thaumatin-like protein | |
| Glyma.04G176200 | / | 3 | ↑ | / | / |  | thaumatin-like protein | |
| Glyma.17G258600 | / | 68 | ↑ | / | / |  | thaumatin-like protein 1-like | |
| Glyma.12G027200+ | 159 | 129 | ↓; ↓ | / | / |  | endoglucanase 5 | |
| Glyma.18G030700+ | / | 35 | ↑ | / | / |  | endo- -beta-glucanase | |
| Glyma.02G269400 | / | 357 | ↓ | / | / |  | endoglucanase 11-like | |
| Glyma.11G101300+ | / | 100 | ↓ | / | / |  | endoglucanase 5-like | |
| Glyma.05G236400+* | / | 477 | ↑ | / | / |  | endoglucanase 8-like | |
| Glyma.08G043600+ | / | 286 | ↑ | / | / |  | endoglucanase 8-like | |
| Glyma.04G111300 | / | 115 | ↑ | / | / |  | endoglucanase 9-like | |
| Glyma.20G171900+ | / | 34 | ↑ | / | / |  | lignin-forming anionic peroxidase | |
| Glyma.20G220900+ | 94 | / | ↑ | / | / |  | germin family protein | |
| Glyma.10G168900 | / | 51 | ↑ | / | 24 | ↑ | germin family protein | |
| Glyma.20G220800 | / | 82 | ↑ | / | 36 | ↑ | germin-like protein subfamily 1 member | |
| Glyma.19G086200 | / | 2 | ↑ | / | / |  | germin-like protein subfamily 1 member 7-like | |
| Glyma.16G061300 | / | 2 | ↑ | / | / |  | germin-like protein subfamily 1 member 7-like | |
| Glyma.11G209400 | / | 283 | ↑ | / | / |  | germin-like protein subfamily 3 member 2 | |
| Glyma.10G071600 | / | 406 | ↑ | / | / |  | germin-like protein subfamily 3 member 2-like | |
| Glyma.11G025800 | / | 30 | ↑ | / | / |  | bon1-associated protein 2-like | |
| Glyma.03G016000 | / | 7 | ↑ | / | / |  | bon1-associated-like protein | |
| Glyma.06G216500 | / | 4 | ↑ | / | / |  | early nodulin-93-like | |
| Glyma.05G009100 | / | 12 | ↑ | / | / |  | early nodulin-93-like | |
| Glyma.05G009000 | / | 167 | ↑ | 47 | / | ↓ | early nodulin-93-like | |
| Glyma.17G117200 | / | 272 | ↑ | 52 | / | ↓ | early nodulin-93-like | |
| Glyma.17G117100 | / | 104 | ↑ | 44 | / | ↓ | early nodulin-93-like | |
| Glyma.09G009800 + | / | 269 | ↑ | / | / |  | early nodulin-like protein 2-like | |
| Glyma.15G049600 | / | 86 | ↑ | / | 85 | ↑ | nodulin 21 -like transporter family isoform 1 | |
| Glyma.17G069200 | / | 136 | ↑ | / | / |  | nodulin 21 -like transporter family protein | |
| Glyma.03G117000 | / | 4 | ↓ | / | / |  | nodulin 21 -like transporter family protein | |
| Glyma.15G211500+ | 191 | 135 | ↑; ↑ | / | / |  | kunitz type trypsin inhibitor | |
| Glyma.16G212100+Ψ | 23 | 20 | ↑; ↑ | / | / |  | kunitz family trypsin and protease inhibitor protein | |
| Glyma.09G163600+Ψ | 14 | 112 | ↑; ↑ | / | / |  | kunitz type trypsin inhibitor miraculin | |
| Glyma.09G163800Ψ | 78 | 63 | ↑; ↑ | / | 180 | ↑ | pathogen-inducible trypsin-inhibitor-like protein | |
| Glyma.16G212200+Ψ | / | 53 | ↑ | / | / |  | kunitz family trypsin and protease inhibitor protein | |
| Glyma.08G342000+ | / | 17,701 | ↑ | / | / |  | kunitz trypsin inhibitor | |
| Glyma.09G163700+Ψ | / | 408 | ↑ | / | / |  | kunitz type trypsin inhibitor miraculin | |
| Glyma.08G341100+ | / | 4,880 | ↑ | / | / |  | kunitz-type trypsin inhibitor kti1-like | |
| Glyma.09G163300+Ψ | / | 17 | ↑ | / | 42 | ↑ | pathogen-inducible trypsin-inhibitor-like protein | |
| Glyma.16G212400Ψ | / | 450 | ↑ | / | 725 | ↑ | pathogen-inducible trypsin-inhibitor-like protein | |
| Glyma.10G184700 | / | 110 | ↑ | / | 99 | ↑ | inhibitor of trypsin and hageman factor-like protein | |
| Glyma.10G184600 | / | 53 | ↑ | / | / |  | inhibitor of trypsin and hageman factor-like protein | |
| Glyma.08G341300+Ψ | / | 154 | ↑ | / | / |  | trypsin inhibitor a-like | |
| Glyma.08G341400+Ψ | / | / |  | / | 331 | ↑ | kunitz trypsin inhibitor | |
| Glyma.08G235400+Ψ | / | / |  | / | 58 | ↑ | kunitz type trypsin inhibitor | |
| Glyma.09G278000+ | 225 | / | ↑ | / | / |  | non-specific lipid transfer protein | |
| Glyma.11G225000+ | 27 | 66 | ↑; ↑ | / | / |  | protease inhibitor seed storage lipid transfer family protein | |
| Glyma.18G033200+ | 50 | 232 | ↑; ↑ | / | 497 | ↑ | protease inhibitor seed storage lipid transfer family protein | |
| Glyma.15G115300 | / | 54 | ↑ | / | / |  | cysteine proteinase inhibitor 1-like | |
| Glyma.09G217800 | / | 20 | ↑ | / | / |  | late embryogenesis abundant protein | |
| Glyma.01G152600 | / | 38 | ↑ | / | / |  | late embryogenesis abundant protein | |
| Ethylene biosynthesis and signalling | | | | | | | |  |
| Glyma.05G222400+* | / | 654 | ↑ | 185 | / | ↓ | 1-aminocyclopropane-1-carboxylate oxidase 1 | |
| Glyma.07G129000+ | / | 374 | ↑ | / | / |  | 1-aminocyclopropane-1-carboxylate oxidase 1 | |
| Glyma.08G029200+ | / | 76 | ↑ | / | / |  | 1-aminocyclopropane-1-carboxylate oxidase 1 | |
| Glyma.09G145000+ | / | 11 | ↑ | / | / |  | 1-aminocyclopropane-1-carboxylate oxidase homolog 1-like | |
| Glyma.09G144700+ | / | 24 | ↑ | / | / |  | 1-aminocyclopropane-1-carboxylate oxidase homolog 1-like | |
| Glyma.17G143900 | 35 | / | ↓ | 5 | / | ↓ | ethylene-responsive transcription factor erf034-like | |
| Glyma.10G016500 | / | 211 | ↑ | / | / |  | ethylene responsive transcription factor 2b | |
| Glyma.20G070100 | / | 5 | ↑ | / | / |  | ethylene-responsive transcription factor | |
| Glyma.10G036700 | / | 27 | ↓ | / | / |  | ethylene-responsive transcription factor 1b-like | |
| Glyma.13G123100 | / | 77 | ↓ | / | / |  | ethylene-responsive transcription factor 1b-like | |
| Glyma.10G061400 | / | 0 | ↓ | / | / |  | ethylene-responsive transcription factor 4-like | |
| Glyma.16G164800 | / | 41 | ↑ | / | 42 | ↑ | ethylene-responsive transcription factor abr1-like isoform x1 | |
| Glyma.13G081600+ | / | 80 | ↓ | / | / |  | ethylene-responsive transcription factor erf012-like | |
| Glyma.13G298600+ | / | 3 | ↓ | / | / |  | ethylene-responsive transcription factor erf053-like | |
| Glyma.05G179900 | / | 48 | ↓ | / | / |  | ethylene-responsive transcription factor erf060-like | |
| Glyma.10G036600 | / | 106 | ↓ | / | / |  | ethylene-responsive transcription factor erf098-like | |
| Glyma.10G223200 | / | 9 | ↑ | / | / |  | ethylene-responsive transcription factor erf110-like | |
| Glyma.20G168500 | / | 19 | ↑ | / | / |  | ethylene-responsive transcription factor erf110-like isoform x1 | |
| Glyma.05G200100 | / | 12 | ↑ | / | / |  | ethylene-responsive transcription factor erf114-like | |
| Glyma.05G157400 | / | 1 | ↓ | / | / |  | ethylene-responsive transcription factor rap2-11-like | |
| Glyma.19G213100 | / | 628 | ↑ | / | / |  | ethylene-responsive transcription factor rap2-12-like | |
| Glyma.10G016501 | / | 162 | ↑ | / | / |  | ethylene responsive transcription factor 2b | |
| Glyma.20G070101 | / | 172 | ↑ | / | / |  | ethylene-responsive transcription factor | |
| Transcription factors | | | | | | | |  |
| Glyma.04G054200Ψ | / | 59 | ↑ | / | / |  | wrky transcription factor 50-like | |
| Glyma.05G160800+Ψ | / | 153 | ↓ | / | / |  | wrky transcription factor 65-like | |
| Glyma.17G097900Ψ | / | 24 | ↓ | / | / |  | wrky transcription factor 72-like | |
| Glyma.18G256500Ψ | / | 34 | ↓ | / | / |  | wrky transcription factor 72-like | |
| Glyma.05G029000 | / | 8 | ↓ | / | / |  | wrky transcription factor 72-like | |
| Glyma.09G240000Ψ | / | 5 | ↓ | / | / |  | wrky transcription factor 72-like | |
| Glyma.19G020600Ψ | / | 11 | ↓ | / | / |  | wrky transcription factor 72-like | |
| Glyma.01G043300 Ψ | / | 5 | ↓ | / | / |  | wrky transcription factor 72-like | |
| Glyma.02G020300 Ψ | / | 4 | ↓ | / | / |  | wrky transcription factor 72-like | |
| Glyma.05G123000+Ψ | / | 13 | ↓ | / | / |  | wrky family transcription factor | |
| Glyma.07G161100+ | / | 3 | ↓ | / | / |  | wrky family transcription factor | |
| Glyma.09G254800Ψ | / | 43 | ↓ | / | 59 | ↑ | wrky transcription factor 22-like | |
| Glyma.15G135600Ψ | / | 36 | ↓ | / | / |  | wrky transcription factor 35 family protein | |
| Glyma.09G029800Ψ | / | 49 | ↓ | / | / |  | wrky transcription factor 35 family protein | |
| Glyma.06G142100Ψ | / | 14 | ↑ | / | / |  | wrky transcription factor 55-like | |
| Glyma.17G119600 | / | 15 | ↑ | / | / |  | myb family transcription factor family protein | |
| Glyma.04G151000 | / | 23 | ↑ | / | / |  | myb family transcription factor family protein | |
| Glyma.03G189600+ | / | 82 | ↑ | / | / |  | myb sant-like dna-binding domain protein | |
| Glyma.06G051900 | / | 4 | ↑ | / | / |  | myb transcription factor | |
| Glyma.08G042100* | / | 12 | ↑ | / | / |  | myb transcription factor myb84 | |
| Glyma.12G195200 | / | 316 | ↑ | / | / |  | myb-like DNA-binding domain | |
| Glyma.02G177800 | / | 2 | ↑ | / | / |  | myb-like dna-binding shaqkyf class protein | |
| Glyma.12G211600 | / | 11 | ↓ | / | / |  | myb-like dna-binding shaqkyf class protein | |
| Glyma.03G051400 | / | 24 | ↓ | / | / |  | myb-like hth transcriptional regulator family isoform 1 | |
| Glyma.14G134700 | / | 4 | ↓ | / | / |  | myb-like protein x-like | |
| Glyma.06G193600+ | / | 27 | ↓ | / | / |  | myb-related protein 306-like | |
| Glyma.02G247100 | / | 22 | ↑ | / | / |  | myb-related protein 308-like | |
| Glyma.14G069100 | / | 37 | ↑ | / | / |  | myb-related protein 308-like | |
| Glyma.09G032100+ | / | 10 | ↑ | / | / |  | transcription factor myb108-like | |
| Glyma.04G051000 | / | 13 | ↑ | 6 | / | ↓ | transcription factor myb1r1-like | |
| Glyma.14G093800 | / | 21 | ↑ | / | / |  | transcription factor myb1r1-like | |
| Glyma.04G036700 | / | 12 | ↓ | / | / |  | transcription factor myb44-like | |
| Glyma.10G142200+ | / | 224 | ↓ | / | / |  | transcription factor myb86-like | |
| Glyma.20G090700+ | / | 181 | ↓ | / | / |  | transcription factor myb86-like | |
| Glyma.19G224600+ | / | 35 | ↓ | / | / |  | transcription factor myb86-like | |
| Glyma.14G201300 | 5 | 9 | ↓; ↓ | / | / |  | transcription factor bhlh61 isoform 1 | |
| Glyma.13G063800 | / | 55 | ↓ | / | / |  | transcription factor bhlh135 | |
| Glyma.19G021400 | / | 241 | ↓ | / | / |  | transcription factor bhlh135 | |
| Glyma.18G258700 | / | 30 | ↓ | / | / |  | transcription factor bhlh135-like | |
| Glyma.09G247000 | / | 20 | ↑ | / | / |  | transcription factor bhlh18-like | |
| Glyma.08G215300 | / | 78 | ↑ | / | / |  | transcription factor bhlh18-like | |
| Glyma.20G153000 | / | 39 | ↑ | / | / |  | transcription factor bhlh36-like | |
| Glyma.02G258300 | / | 39 | ↑ | / | / |  | transcription factor bhlh51-like | |
| Glyma.14G053900 | / | 15 | ↓ | / | / |  | transcription factor bhlh61 isoform 1 | |
| Glyma.16G017700 | / | 14 | ↑ | / | / |  | transcription factor bhlh67-like | |
| Glyma.10G257400 | / | 7 | ↓ | / | / |  | transcription factor bhlh84-like | |
| Glyma.17G236100 | / | 60 | ↓ | / | / |  | transcription factor bhlh85-like | |
| Glyma.14G088400 | / | 16 | ↓ | / | / |  | transcription factor bhlh85-like | |
| Glyma.04G04G054200+ | / | 7 | ↓ | / | / |  | transcription factor bhlh85-like | |
| Glyma.04G172500 | / | / |  | 84 | / | ↓ | transcription factor bhlh135-like | |
| Glyma.20G033300 | / | 11 | ↑ | / | / |  | nac domain protein | |
| Glyma.07G229100+ | / | 20 | ↑ | / | / |  | nac domain protein | |
| Glyma.15G254000 | / | 118 | ↓ | / | / |  | nac domain protein | |
| Glyma.08G173400 | / | 180 | ↓ | / | / |  | nac domain protein | |
| Glyma.02G109800+ | / | 33 | ↓ | / | / |  | nac domain protein | |
| Glyma.12G149100 | / | 6 | ↑ | / | / |  | nac domain protein nac3 | |
| Glyma.12G022700 | / | 140 | ↑ | / | / |  | nac domain protein nac6 | |
| Glyma.09G235700 | / | 5 | ↓ | / | / |  | nac domain-containing protein 100-like | |
| Glyma.16G130200+ | / | 40 | ↑ | / | / |  | nac domain-containing protein 7-like | |
| Glyma.16G069300 | / | 31 | ↑ | / | / |  | nac domain-containing protein 8-like | |
| Glyma.06G248900 | / | 9 | ↑ | / | / |  | nac transcription factor | |
| Glyma.20G033300 | / | 8 | ↑ | / | / |  | nac domain protein | |
| Glyma.13G279900 | / | / | ↓ | / | 105 | ↑ | nac transcription factor | |
| Glyma.04G084900 | / | 27 | ↓ | / | / |  | gata transcription factor 12-like | |
| Glyma.11G068700 | / | 56 | ↑ | / | / |  | gata transcription factor 18-like | |
| Glyma.02G035400 | / | 120 | ↑ | / | / |  | gata zinc finger domain-containing protein 25-like | |
| Glyma.U013300 |  | 30 | ↑ | / | / |  | platz transcription factor family protein | |
| Glyma.01G078600 |  | 38 | ↑ | / | / |  | platz transcription factor family protein | |
| Secondary metabolism | | | | | | | |  |
| Glyma.13G208000+ | / | 328 | ↑ | / | 352 | ↑ | phenylalanine ammonia-lyase EC:4.3.1.24 | |
| Glyma.20G159300+ | / | 172 | ↑ | / | / |  | 4-coumarate-- ligase-like 10-like EC:6.2.1.12 | |
| Glyma.20G114200*+ | / | 236 | ↑ | / | / |  | trans-cinnamate 4-monooxygenase EC:1.14.13.11 | |
| Glyma.11G054500+ | / | 206 | ↓ | / | / |  | caffeoyl- o-methyltransferase EC:2.1.1.104 | |
| Glyma.05G147000 | / | 34 | ↑ | / | / |  | caffeoyl- o-methyltransferase EC:2.1.1.104 | |
| Glyma.20G241700* | / | 801 | ↑ | / | / |  | chalcone isomerase EC:5.5.1.6 | |
| Glyma.05G021900+ | 46 | 28 | ↑; ↑ | / | / |  | flavonoid 3 –hydroxylase EC:1.14.13.21 | |
| Glyma.02G158700 | / | 701 | ↓ | / | / |  | dihydroflavonol 4-reductase EC:1.1.1.219 | |
| Glyma.05G088100+ | / | 29 | ↑ | / | / |  | flavonol synthase EC:1.14.11.23 | |
| Glyma.10G204800+ | 143 | / | ↑ | / | / |  | leucoanthocyanidin reductase EC:1.17.1.3 | |
| Glyma.08G062000 | / | 3 | ↑ | / | / |  | anthocyanidin reductase EC:1.3.1.77 | |
| Glyma.12G197400 | / | 13 | ↑ | / | / |  | terpene synthase EC:4.2.3.19 | |
| Glyma.13G285200 | / | 9 | ↑ | / | / |  | terpene synthase metal-binding domain protein EC:4.2.3.19 | |
| Sugar metabolism | | | | | | | |  |
| Glyma.05G022600+ | / | 154 | ↑ | / | / |  | uridine diphosphate glucose dehydrogenase EC:1.1.1.22 | |
| Glyma.19G212800* | / | 1,909 | ↑ | / | / |  | sucrose synthase EC:2.4.1.13 | |
| Glyma.14G029100 | / | 25 | ↑ | / | / |  | probable sucrose-phosphate synthase 3-like EC:2.4.1.14 | |
| Glyma.13G092500 | / | 49 | ↑ | / | / |  | probable -trehalose-phosphate synthase EC:2.4.1.15 | |
| Glyma.12G051800* | / | 305 | ↑ | / | / |  | pfkb family carbohydrate kinase EC:2.7.1.4 | |
| Glyma.17G242400 | / | 30 | ↑ | / | / |  | alpha amylase family protein EC:3.2.1.1 | |
| Glyma.20G133900 | / | 196 | ↑ | / | / |  | probable beta-d-xylosidase 5-like EC:3.2.1.21 | |
| Glyma.15G024600*+ | / | 70 | ↑ | / | 77 | ↑ | cell-wall invertase EC:3.2.1.26 | |
| Glyma.18G301200 | / | 40 | ↑ | / | / |  | bidirectional sugar transporter n3-like | |
| Glyma.08G360400 | / | 34 | ↑ | / | / |  | bidirectional sugar transporter n3-like | |
| Glyma.04G198400* | / | 47 | ↑ | / | / |  | bidirectional sugar transporter sweet10-like | |
| Glyma.04G238100* | / | 79 | ↑ | / | / |  | bidirectional sugar transporter sweet3-like | |
| Glyma.06G125800 | / | 707 | ↑ | / | / |  | bidirectional sugar transporter sweet3-like | |

Selection of representative differentially expressed genes modulated by inoculation with non-pathogenic and pathogenic isolates of *F. oxysporum*. The values of normalized counts (NC) for the inoculated samples are reported in each interaction (FO36 and FO40) and at each time point (72 and 96 hpi). Induction and repression of each gene in response to pathogen are indicated with up and down arrows, respectively. ^†^The symbol / means that the gene was not found differentially expressed for that time point and strain. Full annotation information can be found in Additional file 4: Table S4. *Expression pattern of these genes were examined by real-time RT-PCR. +Gene was significantly enriched in a GO category based on a Fisher’s exact test with Bonferroni correction for multiple testing. ΨGene was significantly enriched in a Mapman bin based on a Wilcox Rank Sum test with Benjamini Hochberg correction for multiple testing..
