# Supplementary material for: Transcriptome profiling of soybean (Glycine max) roots challenged with pathogenic and non-pathogenic isolates of Fusarium oxysporum
Source: BMC Genomics. 2015 Dec 21;16:1089. doi: 10.1186/s12864-015-2318-2 (PMC4687377; doi:10.1186/s12864-015-2318-2)
Supplement: Additional file 10: Figure S1. — Comparison of RNA-Seq and real-time RT-PCR analysis of selected soybean genes in response to inoculation with F. oxysporum. Expression profiles of (A) carbohydrate kinase (Glyma.12G051800), (B) cell-wall invertase (Glyma.15G024600), (C) bidirectional sugar transporter sweet10-like (Glyma.04G198400), (D) chalcone isomerase (Glyma.20G241700), (E) chitinase (Glyma.01G160100), (F) glutathione s-transferase (Glyma.08G174900), (G) laccase-7-like (Glyma.U027300), (H) myb transcription factor myb84 (Glyma.08G042100), (I) pathogenesis-related protein class 10 (Glyma.15G145600), (J) sucrose synthase (Glyma.19G212800), (K) endoglucanase (Glyma.05G236400), (L) trans-cinnamate 4-monooxygenase (Glyma.20G114200), (M) proline-rich protein (Glyma.15G199700), (N) bidirectional sugar transporter sweet3-like (Glyma.04G238100), (O) 1-aminocyclopropane-1-carboxylate oxidase 1 (Glyma.05G222400), (P) seed linoleate 9 s-lipoxygenase (Glyma.08G189600), (Q) peroxidase (Glyma.09G023000), (R) sulphate transporter (Glyma.15G052000). Dotted lines and histograms represent values expressed as fold change of transcript levels in the inoculated FO36 and FO40 F. oxysporum samples with respect to the transcript levels in control samples at 72 and 96 hpi assessed by RNA-Seq and real-time RT-PCR analysis, respectively. The asterisk (*) means that the FC revealed by RNA-Seq method for that gene is included among HDEGs. (PPTX 149 kb) [file 12864_2015_2318_MOESM10_ESM.pptx]

## Slide 1
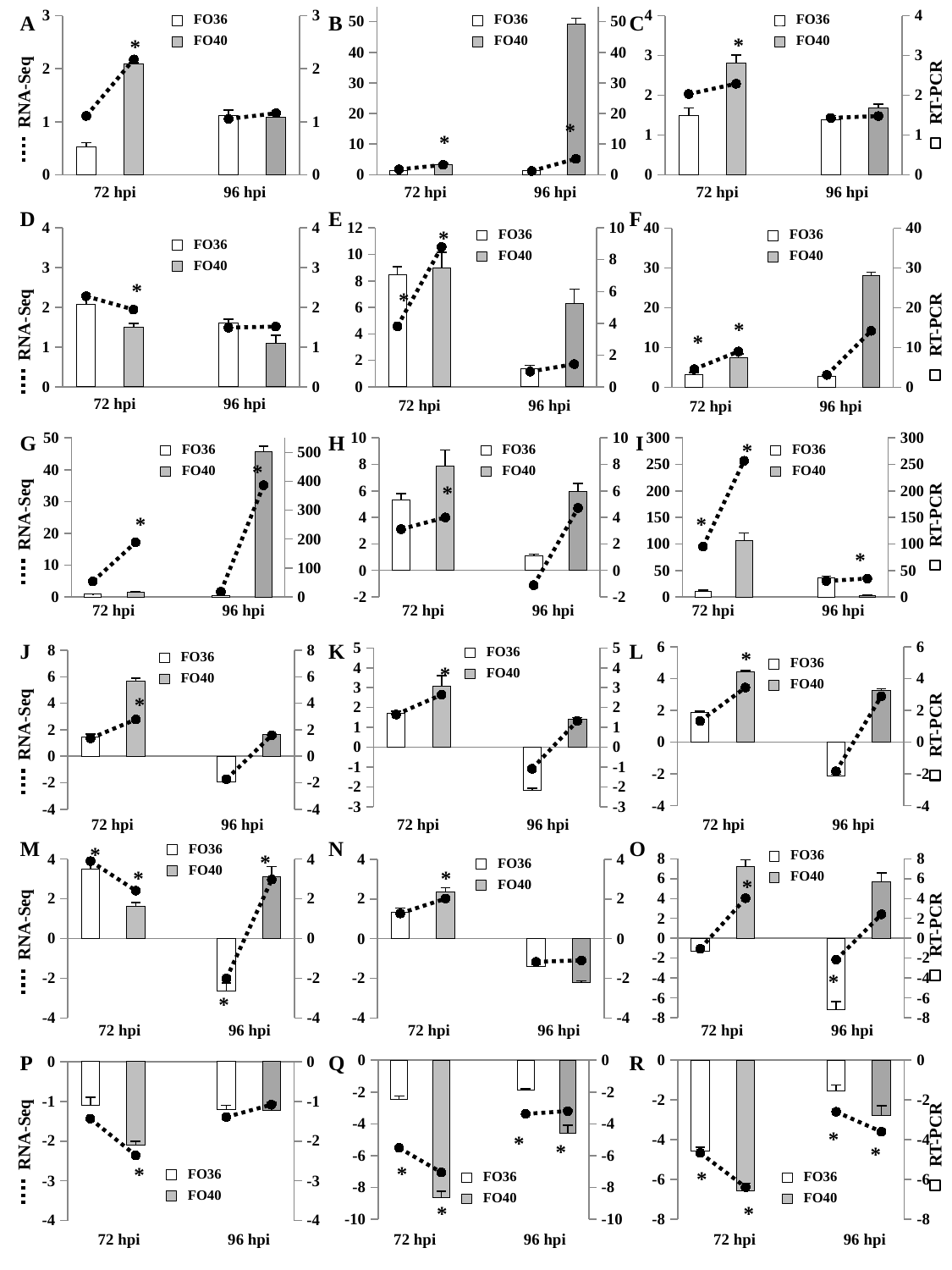

RT-PCR
### Chart
| Category | | |
|---|---|---|
| FO36 | 0.517632461920689 | 1.1093023255814 |
| FO40 | 2.084931521682241 | 2.17577197149644 |
| | None | None |
| FO36 | 1.11728713807222 | 1.051875 |
| FO40 | 1.0792282365044295 | 1.15923566878981 |
### Chart
| Category | | |
|---|---|---|
| FO36 | 1.4742692172911034 | 1.74242424242424 |
| FO40 | 3.2042795103584876 | 3.21538461538462 |
| | None | None |
| FO36 | 1.3013418554419327 | 1.21153846153846 |
| FO40 | 49.18000580121629 | 5.19607843137255 |
### Chart
| Category | | |
|---|---|---|
| FO36 | 1.4845235706290456 | 2.03174603174603 |
| FO40 | 2.8088897514759914 | 2.29032258064516 |
| | None | None |
| FO36 | 1.3851094681109235 | 1.42982456140351 |
| FO40 | 1.681792830507429 | 1.47619047619048 |A
B
C
FO36
FO40
FO36
FO40
FO36
FO40
RNA-Seq
*
*
*
*
72 hpi
96 hpi
72 hpi
96 hpi
72 hpi
96 hpi
D
E
F
### Chart
| Category | | |
|---|---|---|
| FO36 | 2.070529847682756 | 2.28129952456418 |
| FO40 | 1.4948492486349365 | 1.94336569579288 |
| | None | None |
| FO36 | 1.6021397551792478 | 1.49075342465753 |
| FO40 | 1.1019051158766138 | 1.51570132588974 |
### Chart
| Category | | |
|---|---|---|
| FO36 | 7.06162397032524 | 4.56756756756757 |
| FO40 | 7.464263932294451 | 10.5555555555556 |
| | None | None |
| FO36 | 1.156688183905288 | 1.15358744394619 |
| FO40 | 5.241573615433455 | 1.71917808219178 |
### Chart
| Category | | |
|---|---|---|
| FO36 | 3.2265670368885 | 4.5625 |
| FO40 | 7.41270449512297 | 9.0 |
| | None | None |
| FO36 | 2.82842712474619 | 3.1 |
| FO40 | 28.05138308211309 | 14.1818181818182 |*
FO36
FO40
FO36
FO40
FO36
FO40
RT-PCR
RNA-Seq
*
*
*
*
72 hpi
96 hpi
72 hpi
96 hpi
72 hpi
96 hpi
G
H
I
### Chart
| Category | | |
|---|---|---|
| FO36 | 9.189586839976254 | 4.85714285714286 |
| FO40 | 16.449821226497015 | 17.1428571428571 |
| | None | None |
| FO36 | 4.316912946017707 | 1.66666666666667 |
| FO40 | 501.4631923645061 | 35.1111111111111 |
### Chart
| Category | | |
|---|---|---|
| FO36 | 5.314743256386033 | 3.11111111111111 |
| FO40 | 7.8898616359468745 | 4.0 |
| | None | None |
| FO36 | 1.11728713807222 | -1.11111111111111 |
| FO40 | 5.979396994539747 | 4.71428571428571 |
### Chart
| Category | | |
|---|---|---|
| FO36 | 10.928322054035158 | 95.0 |
| FO40 | 106.89125368683116 | 257.0 |
| | None | None |
| FO36 | 36.00187151004186 | 30.0 |
| FO40 | 2.5315131879405657 | 34.7857142857143 |RT-PCR
*
RNA-Seq
FO36
FO40
FO36
FO40
FO36
FO40
*
*
*
*
*
72 hpi
96 hpi
72 hpi
96 hpi
72 hpi
96 hpi
J
K
L
### Chart
| Category | | |
|---|---|---|
| FO36 | 1.8531761237807425 | 1.33175355450237 |
| FO40 | 4.407620463506434 | 3.43689320388349 |
| | None | None |
| FO36 | -2.12 | -1.83783783783784 |
| FO40 | 3.2716082342311266 | 2.88528678304239 |
### Chart
| Category | | |
|---|---|---|RT-PCR
FO36
FO40
### Chart
| Category | | |
|---|---|---|*
FO36
FO40
RNA-Seq
FO36
FO40
*
*
72 hpi
96 hpi
72 hpi
96 hpi
72 hpi
96 hpi
M
N
O
FO36
FO40
*
RT-PCR
FO36
FO40
*
RNA-Seq
### Chart
| Category | | |
|---|---|---|
| FO36 | -1.35 | -1.0531914893617 |
| FO40 | 7.210003700886666 | 4.03909465020576 |
| | None | None |
| FO36 | -7.16 | -2.15549597855228 |
| FO40 | 5.696200782388279 | 2.40725126475548 |
### Chart
| Category | | |
|---|---|---|
| FO36 | 3.5064228852641373 | 3.89513108614232 |
| FO40 | 1.613283518444254 | 2.40816326530612 |
| | None | None |
| FO36 | -2.63 | -2.0078431372549 |
| FO40 | 3.1166583186420005 | 2.97214854111406 |
### Chart
| Category | | |
|---|---|---|
| FO36 | 1.3379275547861103 | 1.27272727272727 |
| FO40 | 2.361985322859058 | 2.01694915254237 |
| | None | None |
| FO36 | -1.4 | -1.16546762589928 |
| FO40 | -2.22 | -1.09090909090909 |FO36
FO40
*
*
*
*
*
72 hpi
96 hpi
72 hpi
96 hpi
72 hpi
96 hpi
P
Q
R
RT-PCR
### Chart
| Category | | |
|---|---|---|
| FO36 | -2.44 | -5.51470588235294 |
| FO40 | -8.63 | -7.05769230769231 |
| | None | None |
| FO36 | -1.89 | -3.37414965986395 |
| FO40 | -4.59 | -3.19736842105263 |
### Chart
| Category | qPCR | RNA-Seq |
|---|---|---|
| FO36 | -4.59 | -4.66666666666667 |
| FO40 | -6.58 | -6.38961038961039 |
| | None | None |
| FO36 | -1.54 | -2.59386973180077 |
| FO40 | -2.8 | -3.6 |
### Chart
| Category | | |
|---|---|---|
| FO36 | -1.1 | -1.43603133159269 |
| FO40 | -2.11 | -2.35964912280702 |
| | None | None |
| FO36 | -1.2 | -1.39563862928349 |
| FO40 | -1.23 | -1.08374384236453 |RNA-Seq
*
*
*
*
*
*
FO36
FO40
*
FO36
FO40
FO36
FO40
*
*
72 hpi
96 hpi
72 hpi
96 hpi
72 hpi
96 hpi
